# Book on the Physician Himself and Things That Concern His Reputation and Success

**Published:** 1893-05

**Authors:** 


BOOK ON THE PHYSICIAN HIMSELF, AND THINGS THAT CONCERN HIS REPUTATION AND SUCCESS. By D. W. Cathell, M. D. New tenth edition (author's last revision). Thoroughly revised, enlarged, and rewritten. In one handsome royal octavo volume, 348 pages, bound in extra cloth. Price, postpaid, $2.00, net. Philadelphia : The F. A. Davis Co., Publishers, 1914–16 Cherry Street.

This well-known book has been reviewed in these pages years ago. It has now passed into its tenth edition, which shows how valuable it has become to the profession. It is a most admirable magazine of advice to the practitioner upon behavior, morals, the observance of the golden rule, the exhibition of tact and all that, as the title-page expresses it, "concerns his reputation and success." It may be called a "Manual of Conduct."

As children we hear much about "conduct." It has made a formidable item in our weekly school report, and it is forever dinned into our ears by our elders. As we grow older and gradually reach the period when we are beyond the accountability to parents, guardians, and teachers, less is heard of the subject until finally all reference to it ceases. Yet the conduct of many of us who have reached the age of responsibility is such that it would seem that we were still in need of admonition upon this very important element of our career. We will not take it from the lips of those who see our faults, and so this book fills the need admirably. Its chapter upon Homœopathy will prove a rather bitter pill to those who practice according to the tenets of our school, but they need not mind that for the sake of the other valuable lessons it teaches.

